# Rumours, myths, and misperceptions as barriers to contraceptive use among adolescent girls and young women in South Africa

**DOI:** 10.3389/frph.2022.960089

**Published:** 2022-09-15

**Authors:** Kim Jonas, Zoe Duby, Kealeboga Maruping, Jane Harries, Catherine Mathews

**Affiliations:** ^1^ Health Systems Research Unit, South African Medical Research Council, Cape Town, South Africa; ^2^ Adolescent Health Research Unit, Division of Child / Adolescent Psychiatry, University of Cape Town, Cape Town, South Africa; ^3^ Division of Social and Behavioural Sciences in the School of Public Health and Family Medicine, University of Cape Town, Cape Town, South Africa

**Keywords:** adolescent girls, contraceptives, contraceptive use, myths, rumours, young women

## Abstract

**Background:**

Rumours, myths, and misperceptions about contraceptives are a barrier to contraceptive use in general, but more so among adolescent girls and young women (AGYW). As rumours and misinformation disseminate easily, it is important to explore how they affect the uptake of contraceptives among AGYW at risk of unintended pregnancies. This study used qualitative methods to explore whether rumours, myths, and misperceptions about contraceptives remain barriers to modern contraceptive use among AGYW who were beneficiaries of a combination HIV prevention intervention in South Africa.

**Methods:**

Four (4) once-off in-depth interviews, 53 serial in-depth interviews, and 19 focus group discussions (FGDs) with 185 AGYW aged 15–24 years living in 5 of the 10 intervention districts were conducted as part of the HERStory 1 Study. Interviews and FGDs were audio recorded and data were analysed thematically, aided by Nvivo 12 software.

**Results:**

Rumours, myths, and misperceptions about contraceptives, as well as sociocultural norms regarding contraception seriously hinder AGYWs’ use of modern contraceptives. Peer/friends’ disapproval and parents’ and boyfriend’s lack of support for AGYWs’ use of contraceptives, based on rumours and perceived side effects, also impede AGYWs’ access and use of contraceptives.

**Conclusion:**

Sexual and reproductive health programmes could address social norms that disapprove of contraception and target rumours, myths, and misperceptions regarding modern contraceptive methods through educational campaigns and community engagements. Promoting the use of contraception in the community and men’s acceptance of contraceptive use, in particular, may increase their understanding of modern contraceptives and, subsequently, their approval for their partners to use them.

## Background

An unmet need for contraception among adolescent girls and young women (AGYW) contributes to high teenage pregnancy rates, which are decreasing at a slower rate in South Africa compared to other low- and middle-income countries (LMICs). In South Africa, about one in five (19%) women of reproductive age (15–49 years) have an unmet need for contraception, with even higher unmet need among adolescent girls aged 15–19 years at 31%, and 28% for young women aged 20–24 years (1). A range of contraceptive methods including oral contraceptives, injectable contraceptives, subdermal implants, contraceptive patches, and barrier methods such as condoms and diaphragms, are provided at no cost at public health services in South Africa. However, AGYW are often offered fewer choices of contraceptive methods compared to older women and given limited explanation of the mechanisms of action and side effects of the different contraceptive methods (2–4). As a consequence, adolescents who use contraception utilise a limited range of methods, with most using injectable contraceptives (70%) followed by the pill (20%) and very few using other methods (5–7). A consequence of inadequate education about contraception is that adolescents report fear of side effects, which is an important factor hindering contraceptive use, especially in LMICs (8).

Sociocultural beliefs, myths, and misinformation about contraceptives are negatively associated with contraceptive use (9–11). Rumours, another concept linked to myths and misperceptions, defined as “unverified and instrumentally relevant information statements in circulation that arise in the context of ambiguity and that function primarily to help people to make sense and manage threat” (12), are also barriers to uptake of health interventions (13). Rumours are neither true nor false, and their authenticity does not need to be proven beyond merely being discussed (14). In public health, understanding the genesis of rumours is critical given the potentially adverse effects rumours may have on health-related behaviours and medical decision-making (15). Rumours about contraception, such as that the contraceptive injection damages the body, negatively affect the access and use of contraceptives (5, 9, 10). Rumours together with myths and misinformation are likely to contribute to low uptake of contraceptives and subsequently the high unmet need and unintended pregnancy rates among AGYW.

*In this paper*, we employ the term “unintended pregnancy” rather than “unplanned pregnancy”; however, we do so with consideration given its problematic nature. “Unplanned pregnancies” have been defined as pregnancies that occur when a woman is using contraceptives or did not wish to become pregnant, with traditional measurements dichotomously classifying pregnancies as intended or not, based on a woman’s intentions before she became pregnant (16, 17). Complexities of intentions, motivation, and desire are often not captured in the dichotomous biomedical/clinical understandings and definitions of “unintended pregnancy.” As such, there is a need to be sensitive and reflective of the reality of people’s lives, with an understanding of the language that AGYW use to describe their lived experiences of pregnancy, their individual subjective experiences, and how they express themselves under these circumstances. Therefore, we use the individual subjective descriptions of the pregnancy experiences and unexpected discoveries where possible and cautiously used the term “unintended pregnancy” to avoid dichotomously classifying their pregnancy.

Reducing the unmet need for contraceptives and unintended pregnancies among AGYW requires improving the availability and accessibility of acceptable sexual and reproductive health (SRH) services that provide comprehensive contraception information and services. Ideally, SRH services would include, among other things, sexual rights education; information on all available contraceptive methods; confidential, non-judgmental, unbiased person-centred contraception counselling and services including explanations of mechanisms of action and side effects; and choice of contraceptive options. Additionally, SRH services would include treatment and prevention of sexually transmitted infections (STIs) including HIV, and information and counselling services about sexuality. If SRH services are available, accessible, and acceptable to AGYW, this is likely to improve knowledge and information about different methods of contraceptives, and subsequently increase contraceptive uptake. While contraception services are primarily delivered within public health facilities in South Africa, with minimal service outreach programmes to communities and schools, the new Integrated School Health Program (ISHP) presented an opportunity to expand access for AGYW in schools. Unfortunately, the full implementation of the ISHP did not take off as intended, and contraceptive services are limited to public health facilities and a few outreach programmes (18). This limits the accessibility of contraception services to AGYW in schools.

To improve and expand access to SRH services for AGYW, a combination HIV prevention intervention was implemented between 2016 and 2019 (https://www.samrc.ac.za/intramural-research-units/HealthSystems-HERStory) (7). The intervention was implemented in 10 South African districts with a high burden of HIV and where AGYW face multiple deprivations, such as limited access to health services, unequal gender-power dynamics, and limited financial resources. It aimed to promote, among other things, access to comprehensive HIV, TB, and SRH services and SRH education for AGYW. Reducing unintended pregnancies among AGYW was one of the five key goals of the combination HIV prevention intervention, and this was to be achieved through SRH education, and linkage and referral to health services. The intervention components comprising SRH education and services aimed to improve AGYW’s contraceptive knowledge, and thereby dispel rumours, myths, and misperceptions relating to contraceptive methods. This study explores whether the existing myths and misperceptions, including rumours about contraceptives, were barriers to the access and use of contraceptives among AGYW who were beneficiaries of the combination HIV prevention intervention in five South African districts (7). Understanding the prevalent rumours, myths, and misperceptions in these communities, which have disproportionately high rates of HIV and teenage pregnancy, is critical in order to inform the design and implementation of interventions.

## Methods

### Study design and setting

This qualitative study was conducted among AGYW living in 5 of 10 ten South African districts in which the combination HIV prevention intervention was being implemented. Five districts were purposively selected, as well as two schools per district from a list of schools where the intervention was being implemented. AGYW were invited to participate in the study by a team of researchers with the assistance of the intervention implementers and school liaison teachers. To obtain further details about the study methods, refer to the HERStory report (7).

For this article, a qualitative descriptive study design was employed to describe the myths and misperceptions around contraception among AGYW.

### Sampling

The sampling framework consisted of districts, schools, and out-of-school clubs where the combination prevention intervention was implemented in five provinces of South Africa. We selected 5 of the 10 districts within five provinces; the selection was based on the representation of the principal recipients (PRs) who were implementing the intervention (at least one PR per district) as well as on the feasibility to conduct the study (e.g., in terms of logistics), among other things. Every district had to be represented by at least one of the PRs; rather than choosing districts that were represented by the same PR, the PRs had to be spread across the different districts. The districts were semi-urban, rural, and urban. A total of 10 schools were selected (two schools within each district). For out-of-school intervention recipients, one Rise Club was purposively selected from each of the five intervention districts. Rise Clubs were constituted by 15–20 young women who met regularly or at least once a month to discuss mainly SRH issues but also general health and education issues that affected them, following a curriculum contained in Rise Magazines. To be eligible to participate in this study, AGYW had to have been a member and attended at least two sessions of SRH-related interventions (see Box 1 for intervention description). Further details about the sampling can be found in the study by Jonas et al. (5) and in the website https://www.samrc.ac.za/intramural-research-units/HealthSystems-HERStory (5, 7).

Box 1. **Description of the combination HIV prevention intervention components**

**Table:** 

Name	Description	Intervention components
Soul-Buddyz Club	An in-school peer education/youth club model in primary schools for children struggling academically, affected by HIV or with signs of neglect. Clubs were facilitated by educators, who attended annual training, and used age-appropriate material	**Biomedical** Linkage and referral to health and other services **Behavioural** SRH education and peer support **Structural** Promote access to grants. Promote an environment for on-going learning Social cohesion
Keeping Girls In-school	A high school-based intervention for adolescent girls at risk of dropping out of school including those affected by HIV, with caregiving responsibilities or with signs of neglect. It aimed to identify and support female learners who were at risk of dropping out of school prematurely. It comprised of a peer education programme facilitated by Peer Group Trainers or Health Educators	**Biomedical** HIV testing; TB, STI, and GBV screening; Linkage and referral to services **Behavioural** SRH education; peer support; home visits to encourage school attendance **Structural** Career guidance; homework support; Promote an environment for on-going learning
RISE clubs (in-school)	Rise clubs are constituted by 15–20 young women from a school, who meet regularly to discuss issues that affect them. The clubs also linked young women to career guidance through career jamborees and homework support. The curriculum is contained in Rise magazines	**Biomedical** Linkage and referral to health services including HCT, PMTCT, ART, SRH **Behavioural** SRH education; caregiving support; peer support; build self-efficacy and resilience **Structural** Social cohesion Community activism Career guidance
RISE Clubs (out-of-school)	The clubs are constituted by 15–20 young women from a community, who meet regularly to discuss issues that affect them. The clubs also linked young women to educational and economic opportunities and local microenterprise development organisations	**Biomedical** Linkage and referral to health services including HTS, PMTCT, ART, SRH services **Behavioural** SRH education; caregiving support; peer support; build self-efficacy and resilience **Structural** Social cohesion Economic strengthening Community activism
Health and welfare jamborees	These events were held in school or community venues and brought health, social, and other services to communities to facilitate access for AGYW and their communities	**Biomedical** HTS; TB, STI, and GBV screening; linkage and referral to health services **Behavioural** SRH education **Structural** Career opportunities; social grants; birth registrations
Community dialogues	Targeted at men and women 14 years of age and above living in the areas of the AGYW intervention. Trained facilitators used promotional materials to guide dialogues in school or community venues. They promoted gender equity, prosocial male norms, and the uptake of men’s SRH services	**Biomedical** Linkage and referral to health services **Behavioural** SRH education **Structural** GBV prevention

TB, tuberculosis; STI, sexually transmitted infections; GBV, gender-based violence; HTS, HIV testing services; PMTCT, prevention of mother-to-child transmission; ART, antiretroviral therapy; SRH, sexual and reproductive health; HCT, HIV counselling and testing.

Within each school, we conducted once-off individual in-depth interviews (IDIs) and focus group discussions (FGDs) with AGYW who participated in the intervention. FGDs were comprised of 6–10 AGYW per group. We considered FGDs to be well suited to younger women as working in a group setting can elicit diverse opinions, beliefs, attitudes, and experiences and encourage others in the group to reflect on or identify with the issues raised. FGD groups were stratified by age group (15–18 and 19–24 years). We also conducted two sets of serial in-depth interviews (SIDIs) with participants in and out of school in each district.

### Participants

AGYW between the ages of 15–24 years who participated in one or more of the key components of the combination intervention were recruited from the schools and the out-of-school clubs within the districts. Participants for the SIDIs were identified from the FGDs as participants that may be able to share more opinions and views outside a bigger group on specific issues surrounding contraception services. Once-off individual in-depth interviews were used when it was not possible to form a group of 6–10 AGYW. Thus, a total of 19 FGDs, 53 SIDIs, and 4 IDIs comprising of 185 AGYW who were intervention recipients were included in this study.

### Data collection tools

An open-ended semi-structured interview guide with probes was used to explore AGYWs’ perceptions of contraception services as means to gain an understanding of their perceived barriers and enablers to accessing the services. The questions about contraceptives and contraception services were part of a broader semi-structured interview guide, which was used to explore a variety of factors related to the intervention and access to health services among AGYW. The broader questions were related to health services access and use; an example: *We are interested in hearing from you about the health services you and your friends receive at school and in the community. Please tell us your views about contraceptives. Probe questions: Do you use contraceptives? Why do you use or not them? What makes it easy or difficult for young women and girls like you to access and use contraceptives? Please elaborate*.

The framing and concept of “unintended” pregnancy was not the focus of investigation in this study and, therefore, we cautiously use the term “unintended,” and where possible, we use the words of AGYW respondents themselves when describing their unexpected discovery of a pregnancy, rather than categorising the pregnancies as “unintended” or not.

All interviews and discussions were conducted between August 2018 and March 2019, in the language preferred by the AGYW (either in English, isiXhosa, isiZulu, or Setswana), audio recorded, and accompanied by hand-written notes taken during the discussions, which lasted 30–60 min. A brief questionnaire to document AGYW socio-demographic characteristics was also administered.

### Procedure

Prior to conducting the group discussions, informed consent was obtained from all participants. We obtained parental/caregiver consent for participants under the age of 18 years and obtained assent from these participants. The interviews and FGDs were conducted by experienced female facilitators within the age groups of 25–35 years of age, with assistance from a female note-taker in the same age group as facilitators. Two researchers (KJ and KM) facilitated the group discussions and conducted the SIDIs and once-off individual in-depth interviews with AGYW. Both facilitators are experienced Black African female researchers with one having obtained a PhD (KJ) and the other one an Honours degree (KM) at the time of data collection. The facilitators and note-taker spoke one or two of the native languages spoken in the intervention districts other than English. The race and languages spoken by the facilitators and that of the AGYW were matched purposely to ensure the data collection team was accepted, trusted, understood, and in turn could understand the nuances of what was said during the interviews. There were no existing relationships with participants prior to data collection for this study and, therefore, no conflict of interest between the participants and the data collection team. Further details about the study procedure are documented in Jonas et al. (5).

Data saturation, where no new topics or issues came up during data collection, was discussed after the 15th FGD by the research team. It became clear that there were no new views transpiring from the discussions by the 19th FGD, and data were deemed saturated at this point with regard to the FGDs. With regard to SIDIs, three interviews were conducted with AGYW with a focus on a specific issue such as contraceptive use. The specific issue was discussed extensively throughout all three interviews until there was no new information emerging about the topic.

### Data analysis

Data were transcribed verbatim from the audio recordings, reviewed by the interviewer for accuracy, translated to English if they were conducted in another language, reviewed again to ensure content was not lost in translation, and then finalised for analysis. Typed transcripts were read, and codes were developed, defined, and refined based on the objectives of the study; thus, themes were derived from the data. A team of independent researchers developed and discussed the coding until they reached consensus. Then thematic analysis was used, and codes were grouped into sub-themes and then into themes. Data were coded using Nvivo 12 qualitative data analysis software. The coded transcripts were analysed by running query reports and primary document tables of codes by theme to explore the issues from the various discussions as shown in Table 1. The study team met regularly to compare and discuss findings until consensus was reached. The reporting of the methods and the results in this study adhered to the Consolidated Criteria for Reporting Qualitative Research (COREQ) (19). We have filled and provided the COREQ as a Supplementary File S1.

**Table 1:** Example of coding process and data synthesis of the rumours, myths, and misperceptions as barriers to contraceptive use among AGYW who were recipients of a combination HIV prevention intervention in five districts of South Africa, HERStory study, 2018–2019.

Overarching theme	Sub-themes	Illustrating examples/quotes
Contraception service benefits	Benefits of using contraceptives	*“It is important because what if you get raped while walking, get kidnapped and raped, there is a difference in what happens because you will be safe from being pregnant even though you can have STI's and HIV.” **AGYW 15–18 years, FGD*** *“Prevention is the way to go because we don’t get pregnant from our boyfriends only, but a situation may occur [rape] where you find yourself raped, so if you are on prevention, you can be protected …. You must remember that when they rape you, they don’t use a condom, so prevention is better because it can protect you from being pregnant.” **AGYW 15–18 years, FGD*** *“You don’t care about what people think [when using contraceptives] so long you know you are going to be safe if you are having sex.” **AGYW 15–18 years, SIDI*** *“They [girls using contraceptive] are doing well because they are protecting their future so that they don’t conceive whilst at school … I think family planning is a right thing … Because if we cannot pursue family planning we would have a lot of kids” **AGYW 19–24 years, SIDI*** *“I think it is wise and important for one to use a condom because if they are sexually active, they can prevent pregnancy and STIs” **AGYW 15–18 years, FGD***
Barriers to contraception services	Perceived general barriers to contraceptive access and use	*“Some clinics can be frustrating like you would go to a clinic for contraception, the nurses will start asking all sorts of question; why are you here? Young as you are! Do you have a boyfriend? And because of these questions and that you feel embarrassed you end up leaving without accessing the services.” **AGYW 15–18 years, FGD*** *“I agree with what she is saying regarding nurses not being able to talk to people nicely, they can’t. A nurse will utter something that will be so hurtful, when you speak and tell them you are here to get contraceptives, they won’t speak to you privately in a room instead they will loudly say why are you here for contraceptives in front of people and you can imagine how many people are at the clinic. They will make noise and say why are you here for contraceptives when you are so young, why? You like boys.” **AGYW 15–18 years, FGD*** *“Ahh! Sometimes when you arrive, you will find that the nurses are mean …. I wanted to do family planning …. Then, they shouted at me, and remarked that I am too young …. I was not comfortable about what happened ever since. So, I accepted that it means I will never go to the clinic … No [they didn’t give me the contraceptives … It was my first time and that was my last day I visited the clinic.” **AGYW 15–18 years, SIDI***
	Myths and misconception	*“… the needle makes other people gain weight, it makes others lose weight. Out there people will start to gossip about you and say you have AIDS, you have TB you are sick and stuff.” **AGYW 15–18 years, FGD*** *“… Some say contraceptives are going to ruin their wombs and in the future, they will not be able to have kids, so they decide not to use contraceptives because they would like to have kids in future.” **AGYW 15–18 years, FGD*** *“… and even when I am ready, I don’t want to use preventative methods because I feel as though … How do I say this … not that it’s not healthy … but it makes you sick. People I know that have prevented are sick, some get thin, some get fat I don’t know, they say it depends on how the injection affects you …” **AGYW 15–18 years, SIDI***
Influence of the combination HIV prevention intervention on contraceptive use and information	AGYW feeling educated and informed about using contraceptives	*“Like contraceptive, I did not know the different types but now I have a little bit of knowledge about them” **AGYW 15–18 years, FGD*** *“In the programme we are taught as girls, how to behave ourselves as well as how to prevent (family planning)…” **AGYW 15–18 years, SIDI***
	Positive influence on overall livelihood of AGYW	*“… it has changed my life because I was not allowed to prevent [use contraceptives], but I joined the programme and told them [parents] that not only a person that sleeps with boys should prevent … a person could get raped, so then they understood.”* ***AGYW 15–18 years, FGD*** *“… The programme has also helped me because (without it) I would have fallen into the trap of falling pregnant and having a child.” **AGYW 15–18 years, FGD*** *“I think it has changed my life because my mom did not want me to prevent, so I joined Rise (Rise Club) and explained to her what they told us there. So she let’s me prevent now …” **AGYW 19–24 years, SIDI***

AGYW, adolescent girls and young women; FGD, focus group discussion; SIDI, serial in-depth interview.

### Trustworthiness

Triangulation of findings from different data sources (AGYW 15–18 years old group, AGYW 19–24 years old group, research assistants’ notes, and interviewers’ observations), and data methods (FGDs, SIDIs, IDIs, and feedback workshops) were used to increase the validity of our findings. Feedback workshops are a method whereby the study results are presented to the participants and/or peers to confirm validity of data interpretations with study participants. In this study, feedback workshops were conducted with study participants and other intervention beneficiaries who did not necessarily participate in the study. Field notes, debriefing sessions, and data collection team reflections between the field team and the larger study team were conducted regularly to discuss themes coded from the data. The transcription–translation process of raw data was performed by the research assistants, reviewed and quality checked by authors or project investigators, and was ultimately reviewed by the authors, keeping an audit trail to help validate the coding process and data analysis.

## Findings

The findings of this study are presented below starting with the brief description of the participants’ characteristics and, thereafter, themes that are presented in three key categories emanating from the data. The quotes are translations of raw data with descriptive details of the participant citing the quotes in bold, indicating the type of discussion (IDI, SIDI, FGD) the participant was part of.

### Participants

A total of 185 AGYW participated in the study with the majority being between the ages of 15–19 years (147, 79.5%). The majority of AGYW (154, 83.2%) were enrolled in school. Among those not in school, 22 (71.0%) had completed secondary school. The majority of AGYW (165, 89.2%) were currently living with at least one parent. Almost all AGYW were receiving and/or living in a household that received a social grant as the main source of financial support (approximately between 29 USD for the child support grant and 111 USD for the pensioners’ grant). Among the AGYW, 49 (26.5%) were taking care of a child or other family members in the household. A small proportion, 38 (20.5%), reported to have ever been pregnant.

### General views about contraceptive use

During the interviews and group discussions, AGYW were asked to share their views on contraceptive use. Many AGYW articulated the belief that using contraceptives was “bad” for the body, while others thought using contraceptives was good for preventing unintended pregnancies. In some cases, AGYW expressed conflicted views, believing that, on the one hand, using contraceptives was good, while at the same time expressing fears of side effects.

*I think it’s not right on one hand, but I think on the other hand it’s right. On the not right side, it’s said that the injection damages the body. **AGYW 15–18 years, FGD***

One concern expressed by AGYW was the fear of being a victim of violence. Stories about forceful removals of the implant from women’s arm by thieves wanting to smoke it have been reported in Cape Town (20). However despite these fears, AGYW acknowledged the benefits of being protected during sex.

*Thieves steal it (the implant) from the train and smoke it … they will cut you … on the right side you can be safe even if your boyfriend say we mustn't use a condom or the condom burst while having sex, so it protects you. **AGYW 15–18 years, FGD***

*Prevention is the way to go because we don’t get pregnant from our boyfriends only, but a situation may occur (rape) where you find yourself raped, so if you are on prevention, you can be protected … You must remember that when they rape you, they don’t use a condom, so prevention is better because it can protect you from being pregnant. **AGYW 15–18 years, FGD***

### Rumours, myths, and misperceptions about contraceptive use

We have categorised the rumours, myths, and misperceptions’ themes about contraceptives into three categories: perceived side effects; perceived ineffectiveness of contraceptives; and peer/friends, partners-related rumours, myths, and misinformation.

#### Perceived side effects related rumours, myths, and misperceptions

Fears about known and perceived side effects were major barriers to contraceptive use reported by AGYW. The most common side effects described by AGYW included weight gain or weight loss, bleeding, absence of menstrual period, and body changes. Majority of AGYW shared the view that contraceptives damage the body, and that their side effects have long-term effects, including fertility issues.

*The injection (contraceptive injection) damages you… there are dangers associated with using contraceptives. **AGYW 15–18 years, FGD***

*Both injection (contraceptive injection) and tablets (oral contraceptives) damage a person inside, you don’t get periods for some time… you can even spend years without having periods. **AGYW 15–18 years, FGD***

#### Perceived ineffectiveness of contraceptives

Most AGYW appeared to lack trust in the effectiveness of contraceptives as a method to prevent pregnancy. The perceived ineffectiveness of contraceptives appears to be based on a lack of accurate information about the mechanism of action of the different contraceptive methods and not necessarily on potential non-adherence. They stated that the contraceptive injection sometimes does not get absorbed in the body stating that it “comes out” without any notice, therefore, deeming it an unreliable method for preventing pregnancy. Some stated that the contraceptives do not always work as one can still become pregnant despite using them.

*Sometimes the injection comes out immediately, without you noticing, then you become pregnant just like that. **AGYW 15–18 years, FGD***

*They say you can sometimes be pregnant and have children whilst on injection. **AGYW 15–18 years, FGD***

#### Peers, friends, and partners-related rumours, myths, and misperceptions

AGYW cited negative perceptions of contraceptives amongst their peers, friends, and partners. Most concerns focused on infertility as a possible consequence of contraceptive use. As a result, AGYW stated that using contraceptives will make them infertile, and therefore chose not to use them. Furthermore, AGYW cited fears about contraceptives making their vagina wet, which they stated made them undesirable to their boyfriends or partners. Condoms were associated with discomfort and irritation from the lubricant, as well as insinuating infidelity to their partners.

*I’m not on contraceptives, but I heard one guy saying he doesn’t like it when his girlfriend is on contraceptives, he said when you use injection, it makes the … vagina wet (others laughing), and then the girl (vagina) becomes boring. **AGYW 15–18 years, FGD***

*I was chilling with my boyfriend and we were having a conversation about contraceptives, and he said that he was afraid of them … his friends say that the needle (injection) will make him infertile. **AGYW 15–18 years, FGD***

### Influence of the combination HIV prevention intervention on contraceptive use and information

Sharing their views on how the intervention programme sessions have impacted on their knowledge and use of contraceptives, most AGYW felt that the intervention had impacted them positively. Many reported feeling educated and informed about contraceptives, and felt they had a good understanding of the importance of avoiding unintended pregnancies. Having this information appeared to have empowered AGYW to discuss SRH-related topics with their parents to allow them to use contraceptives. Some reported that the intervention helped them to be more responsible and choose contraception instead of having children while they are still in school.

*It (participating in intervention) has changed my life because I was not allowed to prevent (use contraceptives), but I joined the programme and told them (parents) that not only a person that sleeps with boys should prevent … a person could get raped, so then they understood. **AGYW 15–18 years, FGD***

*The programme has also helped me because (without it) I would have fallen into the trap of falling pregnant and having a child. **AGYW 15–18 years, FGD***

*In the programme we are taught as girls, how to behave ourselves as well as how to prevent (family planning). **AGYW 15–18 years, SIDI***

## Discussion

This study sought to explore prevalent rumours, myths, and misperceptions about contraceptives among AGYW who were recipients of a combination HIV prevention intervention in five South African districts. Findings suggest that overall, rumours, myths, and misperception serve as barriers to the access and use of contraceptives among AGYW. The perceived side effects of modern contraceptives, especially injectable contraceptives, appear to be the major barrier to contraceptive access and use among AGYW. Research shows that societal and cultural preferences are important factors contributing to the fear of contraceptives’ side effects. Women fear that contraceptives will make their vaginas wet, and negatively affect their sexual relationships with their partners, due to their belief in men’s preference for dry sex (21, 22).

Negative myths and misperception about contraceptives are prevalent among young women and continue to be a barrier to contraceptive use. In this study, most AGYW perceived contraceptive use as “bad” for the body, a view that is likely to be based on the myths and misperceptions around contraception. AGYW also reported lack of trust in contraceptives to effectively prevent pregnancy. It appears that these negative myths and misperceptions stem from poor understanding of the mechanism of action and fear of the perceived side effects of contraceptives. In a previous study, Jonas et al. (5) found that myths and misconceptions around the “side effects” of the contraceptive injection, in particular, were one of the barriers to the access and use of contraceptives by AGYW (5). Research in sub-Saharan Africa has also highlighted the influence of belief in rumours, myths, and misperceptions about contraceptive methods on young women’s contraceptive use (4, 5, 23–25). This is concerning as the rates of unintended pregnancies among this sub-population are declining at a slower rate particularly in South Africa. This highlights the need to intensify interventions to improve accurate knowledge and information about modern contraceptives, such as through educational campaigns. These interventions may help debunk the rumours, the negative myths, and misperceptions about contraceptives among AGYW and promote access and use of contraception services.

Another important finding from this study is the influence of peers/friends, social networks, and parents’ and boyfriends’ support, or lack thereof, for AGYWs’ use of contraceptives. Some AGYW stated that they are not using contraceptives because they have heard rumours in the community, from friends and peers, that contraceptives are bad for the body, centring on fears relating to infertility. Fears around infertility have long been a salient serious issue when it comes to SRH programmes in sub-Saharan Africa and impede public health interventions to promote contraceptive use among women of reproductive age (13, 26). In general, AGYW trust people closer to them than service providers, such as family members, friends, and peers, and therefore, it is not surprising that negative views and perceived side effects from their friends’, peers’, parents’, and boyfriends’ perspectives influence their opinions and hinder their access and use of contraceptives. From their partners’ perspectives, some AGYW stated that their boyfriends do not want them to use contraceptives as they are suggested to have negative consequences for both male and female reproductive health. Some AGYW reported that their partners suggest that contraceptives will make them, the boyfriend, infertile, suggesting that men may feel some sense of their power being threatened by women’s contraceptive use. This is not surprising as previous research showing that men’s lack of support for their female partners to use contraceptive was one of the reasons women were not using or discontinued contraception (5, 7). This finding suggests that in addition to involving men in SRH services, there is a need to promote men’s and boys’ acceptance of contraception in order to improve AGYWs’ contraceptive use. Widespread belief in the negative myths and misperceptions about contraceptives by AGYW is associated with the lower likelihood of AGYW reporting contraceptive use or discontinuing use (7, 23, 27). While approval of peers/friends, support from parents and boyfriends for the use of contraceptives has been shown to positively influence AGYWs’ access and use of contraceptives (5, 27).

Regarding the intervention’s impact on AGYWs’ SRH knowledge and views on contraceptives, participation in the intervention appeared to have had a positive impact. However, some AGYW still felt they need more information about contraceptives. Some AGYW stated that they were not using contraceptives despite participation in the intervention, suggesting that the intervention alone was insufficient to change some of the views and negative perceptions about contraceptives and convince AGYW to use them.

There are some important limitations in interpreting the findings of this study. First, we did not explore AGYW’s religious or cultural affiliations, which are likely to have a great influence on AGYW’s perceptions of contraceptive use. Further research may need to explore this aspect to get more insights into the influence of religion and culture on AGYWs’ perceptions of contraceptives. Second, this study was conducted during the second and third years of intervention implementation, and the intervention may not have had time to affect the views and perceptions of contraceptive use and other use of SRH services. In-depth exploration of the extent to which the intervention provided education and information to demystify the myths and misperceptions about contraceptives were beyond the scope of this study. Despite these limitations, our findings emphasise the critical importance of addressing negative myths and misperceptions about contraceptive use among AGYW and offer other approaches to promote and increase the use of contraceptives by young people in South Africa.

## Conclusion

Our findings indicate that rumours, myths, and misperceptions about contraceptives persisted as barriers to contraceptive use among AGYW who were beneficiaries of a combination HIV prevention intervention in South Africa. The package of HIV prevention interventions has the potential to improve contraceptive use among AGYW if it can be enhanced with a comprehensive contraception education and counselling component along with general SRH education and awareness to ensure wider coverage of the intervention. SRH interventions need to include efforts to address social norms that disapprove of contraception, and work to debunk myths and misperceptions regarding modern contraceptive methods through educational campaigns and community engagements. Rumours, myths, and misperceptions are very context specific, and therefore qualitative research is crucial in order to describe, unpack, and shed light on contextual sociocultural specificities, so that we can best understand how they impact on contraceptive uptake, use, and acceptability. Promoting contraception at the community level, specifically addressing men’s and boys’ acceptance of contraceptive use by AGYW, may improve acceptability of modern contraceptives and subsequently enable female partners/girlfriends to use them. Testimonies from satisfied contraceptive users in communities could be another strategy to mitigate fears related to contraceptive use among AGYW. Additionally, health workers need to be trained to provide more comprehensive, person-centred, youth-friendly contraception counselling to AGYW when they access SRH services. These interventions may help dispel the existing myths and misperceptions about contraceptive use, improve contraceptive acceptance and approval, and subsequently increase contraceptive use by AGYW.

## Data Availability

The original contributions presented in the study are included in the article/**Supplementary Material**, further inquiries can be directed to the corresponding author.
