# Supplementary material for: Rumours, myths, and misperceptions as barriers to contraceptive use among adolescent girls and young women in South Africa
Source: Front Reprod Health. 2022 Sep 15;4:960089. doi: 10.3389/frph.2022.960089 (PMC9673823; doi:10.3389/frph.2022.960089)
Supplement: Supplementary file 1 [file DataSheet1.pdf]

Consolidated criteria for reporting qualitative studies (COREQ): 32-item checklist

Article: Rumours, myths, and misperceptions as barriers to contraceptive use among adolescent girls and young women in South Africa

YOU MUST PROVIDE A RESPONSE FOR ALL ITEMS. ENTER N/A IF NOT APPLICABLE

**No. Item**	**Guide questions/description**	**Reported on Page #**
**Domain 1: Research team and reflexivity**		
Personal Characteristics		
1. Inter viewer/facilitator	Which author/s conducted the inter view or focus group?	Methods, page 6
2. Credentials	What were the researcher's credentials? E.g. PhD, MD	Methods, page 6
3. Occupation	What was their occupation at the time of the study?	Methods, page 6
4. Gender	Was the researcher male or female?	Methods, page 6
5. Experience and training	What experience or training did the researcher have?	Methods, page 6
Relationship with participants		
6. Relationship established	Was a relationship established prior to study commencement?	Methods, page 6
7. Participant knowledge of the interviewer	What did the participants know about the researcher? e.g. personal goals, reasons for doing the research	N/A
8. Interviewer characteristics	What characteristics were reported about the interviewer/facilitator? e.g. Bias, assumptions, reasons and interests in the research topic	Methods, page 6
**Domain 2: study design**		
Theoretical framework		
9. Methodological orientation and Theory	What methodological orientation was stated to underpin the study? e.g. grounded theory, discourse analysis, ethnography, phenomenology, content analysis	Methods, page 4-5
Participant selection		
10. Sampling	How were participants selected? e.g. purposive, convenience, consecutive, snowball	Methods, page 5
11. Method of approach	How were participants approached? e.g. face-to-face, telephone, mail, email	Methods, page 5
12. Sample size	How many participants were in the study?	Results, page 8

13. Non-participation	How many people refused to participate or dropped out? Reasons?	None
Setting		
14. Setting of data collection	Where was the data collected? e.g. home, clinic, workplace	Methods, page 5
15. Presence of non-participants	Was anyone else present besides the participants and researchers?	Methods, page 5
16. Description of sample	What are the important characteristics of the sample? e.g. demographic data, date	Results, page 8
Data collection		
17. Interview guide	Were questions, prompts, guides provided by the authors? Was it pilot tested?	Methods, page 5-6
18. Repeat interviews	Were repeat interviews carried out? If yes, how many?	N/A
19. Audio/visual recording	Did the research use audio or visual recording to collect the data?	Methods, page 5-6
20. Field notes	Were field notes made during and/or after the inter view or focus group?	Methods, page 5
21. Duration	What was the duration of the inter views or focus group?	Methods, page 5
22. Data saturation	Was data saturation discussed?	Methods, page 7
23. Transcripts returned	Were transcripts returned to participants for comment and/or correction?	N/A
**Domain 3: analysis and findings**		
Data analysis		
24. Number of data coders	How many data coders coded the data?	Methods, page 7
25. Description of the coding tree	Did authors provide a description of the coding tree?	N/A
26. Derivation of themes	Were themes identified in advance or derived from the data?	Methods, page 7, Results, page 8
27. Software	What software, if applicable, was used to manage the data?	NVivo, page 7
28. Participant checking	Did participants provide feedback on the findings?	Methods, page 8
Reporting		
29. Quotations presented	Were participant quotations presented to illustrate the themes/findings? Was each quotation identified? e.g. participant number	Results, page 8-12
30. Data and findings consistent	Was there consistency between the data presented and the findings?	Results, page 11-19
31. Clarity of major themes	Were major themes clearly presented in the findings?	Results, page 8-12
32. Clarity of minor themes	Is there a description of diverse cases or discussion of minor themes?	Results, page 8-12

Once you have completed this checklist, please save a copy and upload it as part of your submission. When requested to do so as part of the upload process, please select the file type: *Checklist*. You will NOT be able to proceed with

submission unless the checklist has been uploaded. Please DO NOT include this checklist as part of the main manuscript document. It must be uploaded as a separate file.

Developed from:

Tong A, Sainsbury P, Craig J. Consolidated criteria for reporting qualitative research (COREQ): a 32-item checklist for interviews and focus groups. *International Journal for Quality in Health Care*. 2007. Volume 19, Number 6: pp. 349 – 357
